# nCoV-2019 infection induced neurological outcome and manifestation, linking its historical ancestor SARS-CoV and MERS-CoV: a systematic review and meta-analysis

**DOI:** 10.1038/s41598-021-92188-x

**Published:** 2021-06-18

**Authors:** Ajay Prakash, Harvinder Singh, Phulen Sarma, Anusuya Bhattacharyya, Deba Prasad Dhibar, Neeraj Balaini, Ritu Shree, Manoj Goyal, Manish Modi, Bikash Medhi

**Affiliations:** 1 Department of Pharmacology, Research Block: B, Post Graduate Institute of Medical Education and Research (PGIMER), Chandigarh, India; 2 Department of Internal Medicine, Post Graduate Institute of Medical Education and Research (PGIMER), Chandigarh, India; 3 Department of Ophthalmology, GMCH, Sector: 32, Chandigarh, India; 4 Department of Neurology, PGIMER, Chandigarh, India

**Keywords:** Neurology, Signs and symptoms

## Abstract

The first systematic review and meta-analysis to help clinician to identify early signs and symptoms of neurological manifestation in COVID-19 positive patients which will further help in early management of patients. Present systematic review and meta-analysis aimed to discuss the prevalence of neurological involvement of the 2019-nCoV patients and assess the symptomatic trend of events as compared to the 2002 “SARS” and 2012 “MERS” pandemics. The articles were systematically screened through several search engine and databases. The articles published or in preprint were included in the study till 15th May 2020. The systematic review done as per the published literatures which included 31 cross sectional, observational studies and case reports which revealed neurological signs and symptoms in SARS-COV-2 disease. For meta-analysis, we included 09 observational and cross-sectional studies which included COVID-19 positive patients and assessed the predominance of various neurological signs and symptoms in COVID-19 patients with relation to SARS-2002 and MERS-2012. Data was analyzed by using the “*MedCalc*” Statistical Software version 19.2.6 and reported as pooled prevalence. Standard I^2^ test was used to analyze the heterogeneity. We have collected and screened about a total 2615articles, finally we have included 31articles for the systematic review and 09 for meta-analysis as per the inclusion/exclusion criteria. The analysis was made as per the prevalence rate of neurological symptoms in COVID-19 positive patients. The cumulative neurological outcome of SARS-2002 and MERS-2012 was assessed to get the trends which was further tried to correlate the events with the current pandemic. During the analysis severity and outcome of neurological manifestations range from simple headache to vague non-focal complaints to severe neurologic impairment associated with seizure or meningitis. Central and peripheral nervous system (CNS/PNS) manifestations were seen during the SARS-2002, MERS-2012 and COVID-19. However, none of the publication had primary or secondary objectives of searching neurological manifestations in the COVID-19 patients and the pathogenic mechanism which will subsequently strengthen the importance to start more prospective clinical trials. The prevalence of neurological signs and symptoms were taken as primary objective. Thereafter, the prevalence of each CNS/PNS symptoms was categorized and their prevalence studied. The selection of Bagheri et al., 2020 may be discussed because they have done the cross-sectional study with the neurological finding and correlated the data with prevalence of the COVID-19 positive patients. The proportion of patients presenting with neurological outcome and clinical/PCR positivity were done. We had searched and followed all the possible online/web source, still the data collection process may remain a limitation of work due to addition of several publications on COVID-19 every day. Due to lack of data of SARS-CoV and MERS-CoV, we have included the case reports, MERS and COVID-19 in CNS/PNS manifestations.

## Introduction

Severe acute respiratory syndrome namely corona-virus 2019 (nCoV-2019) pandemic has changed the understanding and viewpoint of viral infection and given a cause to think more critically in the management of viral infection. β-type SARS-CoV-2 is the version 7.0 of corona virus familyCOVID-19 may be asymptomatic or have mild to severe pneumonia like syndrome^1,2^. Several researches have recommended that 2019-nCoV is one of the predominant viruses that specifically target the human respiratory system. SARS, MERS and COVID-19 essentially exhibit as respiratory distress, which can manifest as mild respiratory symptoms to serious respiratory distress syndrome (ARDS) and incidentally adjunct to gastrointestinal appearances^2^, cardiovascular and neurological involvement^3^. Neurological potentiality is never been studies in any of the published literatures as a primary or secondary objective. If we see the history of the corona virus family and its pandemic situations, it appeared in 2002 as SARS and MERS in 2012. Current ongoing high mortality rate of the present nCoV-2019 as compared to the other counterparts signifies that the pathogenesis is likely completely different from its old counterparts and yet to be understood^4^.

The common symptoms of nCoV-2019 infection starts in 4–5 days with mild fever, mild to moderate cough, running nose and fatigue whereas some other symptoms namely headache, hemoptysis, and dyspnea were reported in several studies. Moderate to severe cases of nCoV-2019 infection may worsen with the development of pneumonia, acute respiratory distress syndrome, acute cardiac problems, and multiorgan failure^4^.

Presently as on June 5, 2020 the total number of coronavirus cases world wide were 6,720,550, with 393,542 deaths (5.85%) and nearly 3,264,238 have recovered till now (48.57%)^5^. This suggests that COVID-19 pandemic is one of the major world public health issue which World Health Organization (WHO) formally declared as pandemic on 11 March 2020 and named as, COVID-19 outbreak^6^. Studies reporting nCoV-2019 infections have found related neurological manifestations (e.g., febrile seizures, convulsions, change in mental status, and encephalitis)^7-9^. Very few reports have described the neurotropic and neuro invasive abilities of corona viruses to humans, which suggest that nasal nCoV-2019 infection, get access to CNS through the olfactory bulb leading to inflammation and demyelination in the bulb^10^. Therefore, current systematic review and meta-analysis aimed to congregate proof on the incidence of CNS/PNS involvement and neurological manifestations in population with COVID-19.

## Methods

### Objectives

Incidence of the neurological manifestations in nCoV-2019 and prevailing prevalence in the context of previous CoV-SARS. Therefore, present study reserves the objectives to find out;

1. Prevalence of neurological outcome in 2019-nCoV infected patients.
2. Correlation of events in SARS, MERS and 2019-nCoV infection.
3. Prevalence of CNS/PNS symptoms in all the corona pandemics.
4. Network analysis of case reports of SARS, MERS and COVID-19.

#### Search methods

The manuscript search was from complete possible online sources. Out of total 2615articles, finally we included 31 articles for the systematic review and meta-analysis. The analysis was made in the two sections, one which is the current nCoV-2019 outbreak for the neurological manifestations and another is cumulative outcome of 2002 and 2012 SARS and MERS outbreak respectively.

#### Database search

Three independent reviewers AJ, HM and PS searched the Google Scholar, MEDLINE (PubMed), EMBASE, OVID, Scopus, Science Direct and unpublished data were screened through MedRixv and BioRixv. The search strategy included both keywords and Medical Subject Headings (MeSH) terms. The keywords used were: “Neuro”, “CNS”, Central nervous System, “anxiety”, “depression’’, “seizure”, “agitation”, “neurological”, “2019-nCov”, 2019 “novel corona virus”, COVID-19, corona virus disease-2019, OR infarction for the corona virus-2 however, all previous studies for SARS-2002 and MERS-2012 where searched with the key words “corona virus- SARS” OR SARS OR SARS-CoV AND “corona virus-MERS OR MERS OR MERS-CoV”. During the screening process we have kept no language restrictions and for articles written in languages other than English, google translate was used to obtain relevant information and extract data if possible, otherwise it was excluded from the analysis. In cases in which the translation cannot work out we have collected data only from the abstract (if it is in English). We retrieved the full text article of the potentially eligible study after screening the title, summary/abstract and type of study as described by search result which met the eligibility criteria for current systematic review and meta-analysis. Databases were systematically searched thoroughly and duplicates were independently screened by authors (DP, NB, AB and HM). In the next phase, articles were selected as per the titles/abstracts published and have the relevance as neurological outcome in COVID-19. For relevant articles, the full texts were obtained and evaluated as per neurological sign and symptoms in nCoV-2019 positive patients. BM, MM, MG and AP were consulted for any discrepancy or confusion. Four authors (HS, DP, RS and NB) have extracted data independently by using pre-tested Cochrane data extraction form.

#### Selection criteria

**Figure 1:**
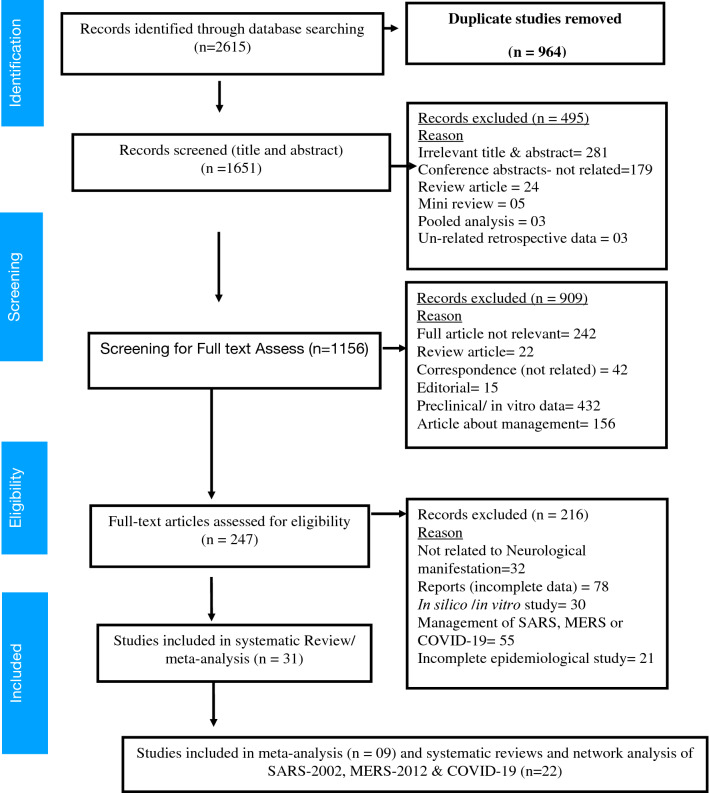
PRISMA chart^2,10–37^ showing study section criteria and process.

The Preferred Reporting Items for Systematic Reviews and Meta-Analyses (PRISMA) and Meta-analysis of Observational Studies in Epidemiology (MOOSE) were followed to analyze and report systematic review and meta-analysis (Fig. 1). The registration of the review protocol was not previously done. The thorough search engine was used to collect all possible studies through Google scholar, MEDLINE (PubMed), EMBASE, OVID, Cochrane Central Library, CNKI, MedRixv and BioRixv and Scopus till June 5, 2020.

#### Statistical analysis

To analyze the differences in neurological impact, levels of anosmia/hyposmia, dyspnea, headache, headache and loss of consciousness were calculated according to the number of respondents per response to the number of total patients as categorical variables. Differences categorical variables were analyzed for proportion analysis by chi-square test. We used network analysis to correlate with the different pandemics namely SARS-2002, MERS-2012 and COVID-19. “MedCalc Statistical Software version 19.2.6 (MedCalc Software bv, Ostend, Belgium; https://www.medcalc.org; 2020)” was used to analyze the data. The fixed and random effect model were used to obtain pooled prevalence. Heterogeneity was investigated (standard χ^2^ test) and represented as I^2^ for the degree of inconsistency. Analyzed report showed both fixed effect and random effect model as necessary as an indication of the variability in the studies. The significant level set at *p* < 0.05 and prevalence range were represented as 95% CI. Publication bias in the selected study was evaluated by plotting the funnel plot and done the analysis accordingly.

## Results

*Inclusion/exclusion criteria of studies* *T*otal 2615 articles were found in databases search and after removing duplicates, title and summary screening articles reduced to 1651, thereafter, a total of 1459 articles were excluded. Full-text screening of the remaining 1156 articles was done. Among these studies, after full-text screening, a total of 31articles were included in the final review (Fig. 1).

*Data collection and analysis*^2,10–37^ A total of 1651 articles were found after preliminary screening of the databases. After title and abstract screening, a total of 495 articles were excluded. Full-text screening of the remaining 1156 articles was done. Among these studies, after full-text screening, a total of 247 articles were included in the final review. The PRISMA flowchart^37^ of the study is shown in Fig. 1, 215 articles were excluded after full-text screen (Not related to neurological manifestation = 32, Reports (incomplete data) = 78, In silico /in vitro study = 30, Management of SARS, MERS or COVID-19 = 55 incomplete epidemiological study = 20). Details of studies with neurological manifestation in SARS-2002, MERS-2012 and COVID-19 summarized in Tables 1, 2 and 3.

**Table 1:** Published observational/cross sectional studies which reported the neurological manifestation to the COVID-19 positive patients.

Author, year	Sample size (SS)	Type of study (ST)	Patient population	Treatment	Neurological condition (NC)	2019-nCoV presence and diagnosis	Type of NS involvement (CNS/PNS)	Remarks
Bagheri et al., 2020^10^	10,069	Cross-sectional study	COVID-19	NA	Anosmia/hyposmia (48.23%)	NA	PNS	Olfactory dysfunction happened in Iran during the COVID-19 epidemic, that correlates with the number of patients infected with COVID-19 across the country
Chen et al., 2020^11^	99	Observational	COVID-19 positive	Oseltamivir, ganciclovir, lopinavir ritonavir, cephalosporins, quinolones, carbapenems, tigecycline	Dyspnea (21 [58.33%] patients), Fatigue (17 [47.22%] patient)	RT-PCR	CNS and PNS	2019-nCoV was of clustering onset, is more likely to infect older men with co-morbidities
Helms, 2020^12^	64	Observational	COVID-19 positive	Midazolam Propofol Sufentanil	Neurological sign (49/58) Agitation (40/58) Confusion (26/58), enhanced tendon reflexes, ankle clonus, and bilateral extensor plantar reflexes (39/58), inattention, disorientation, or poorly organized movements (15/45) Cerebral ischemic stroke (3/13) Encephalopathy (1/8)	RT-PCR CSF	CNS and PNS	At the initial stage author have unable to decide the features of critical illness-related encephalopathy, cytokines, or the effect or withdrawal of medication, and which features were specific to SARS-CoV-2 infection
Huang et al., 2020^2^	41		COVID-19 positive	Remdesivir Lopinavir and Ritonavir Corticosteroid	Headache (3/38), Dyspnea (22/40), Myalgia (18/41)	RT-PCR	CNS and PNS	The neurological sign and symptoms appear in around 3% to 44% of patients
Huang et al., 2020^13^	36	Observational	COVID-19 positive	Oseltamivir, Ganciclovir, Ribavirin Orumifenovirhydrochloride (35/36)	Shortness of breath (21/36), Dyspnea (14/36) Disturbance of consciousness (8/36), Fatigue (17/36) Myalgia (1/36)	RT-PCR	CNS and PNS	Reported most non-survivors are older men with comorbidities conditions especially cardiovascular diseases and COPD
Lechien et al., 2020^14^	417	Observational	COVID-19 positive	NA	Anosmic (284/417) Hyposmic (73/417) Dyspnea (54/417)	Nasopharyngeal swab test (RT-PCR	CNS	Suggested to report anosmia or ageusia as important symptoms of the COVID-19 infection
Mao et al., 2020^15^	214	Observational	COVID-19 positive	NA	Neurological sign (78/214) Headache (28/214), Dizziness (36/ 214), Impaired consciousness(16/78), ataxia, acute cerebrovascular disease (6/78) and Epilepsy Peripheral nervous system (PNS) symptoms Skeletal muscular symptoms (23/78) Hypogeusia (12/214) Hyposmia (11/214)	Head CT, Swab test	CNS and PNS	Non-severe severe COVID-19 patients reported neurologic symptoms manifested as acute cerebrovascular diseases, consciousness impairment and skeletal muscle symptoms
Wang et al., 2020^16^	138	Observational	COVID-19 positive	Oseltamivir Methylprednisolone Azithromycin Moxifloxacin, Ceftriaxone	Headache(9), Dizziness (13), Dyspnea (5)	RT-PCR	CNS	Identified common sign and symptoms at COVID-19 onset were fever, dry cough, myalgia, fatigue, dyspnea, and anorexia
Wang et al., 2020^17^	69	Observational	COVID-19 positive	oxygen support, Corticosteroids, Moxifoxacin	Dyspnea (15/69), Dysgeusia (5/69), Ageusia (1/69), Hyposmia (3/69), Dysgeusia and hyposmia (2/69), Dysgeusia and anosmia (2/69), Ageusia and hyposmia (2/69), Ageusia and anosmia (5/69)	Nasopharyngeal swab test (RT-PCR	CNS and PNS	Older patients or those with underlying comorbidities are at higher risk of death

*NA* not available, *NS* nervous system, *CNS* central nervous system, *PNS* peripheral nervous system, *CSF* cerebrospinal fluid, *RT-PCR* reverse transcription polymerase chain reaction.

**Table 2:** Published case reports of neurological manifestations in COVID-19 positive patients.

Author, year	Sample size (SS)	Type of study (ST)	Patient population	Treatment	Neurological condition (NC)	2019-nCoV presence and diagnosis	Type of NS involvement (CNS/PNS)	Remarks
Duong et al., 2020^18^	01	Case report	COVID-19	Ceftriaxone and Vancomycin, Acyclovir, HCQ	Meningoencephalitis	PCR (CSF-NA)	CNS	They have observed a COVID-19 infection presenting as an isolated meningoencephalitis without respiratory involvement
Karimi et al., 2020^8^	01	Case report	COVID-19 positive	Chloroquine and Lopinavir-ritonavir	GTC convulsion	Brain MRI	CNS	Association between frequent seizures and COVID-19 may be due to encephalitis and the invasion of the virus to the brain or toxic effect of inflammatory cytokines
Moriguchi et al., 2020^21^	01	Case report		Laninamivir and antipyretic	Meningitis/encephalitis		CNS	Specific SARS-CoV-2 RNA was not detected in the nasopharyngeal swab, it was detected in CSF
Paniz-Mondolfi et al., 2020^22^	01	Case report	COVID-19 positive	HCQ and enoxaparin	Confusion Agitation and combative	Nasopharyngeal swab test (RT-PCR positive)	CNS	Transmission electron microscopy of sections obtained at post-mortem examination revealed the presence of 80–110 nm viral particles in frontal lobe brain sections
Scheidl et al., 2020^23^	01	Case report	COVID-19 positive	NA	Guillain Barre syndrome	RT-PCR positive	PNS	Our case draws attention to the occurrence of GBS also in patients with COVID-19 (coronavirus disease 2019), who did not experience respiratory or general symptoms
Sedaghat et al., 2020^24^	01	Case report	COVID-19 positive	HCQ, LPV/RTV Azithromycin	Guillain Barre syndrome	RT-PCR positive	PNS	Suggested the treatment with IVIG or plasmapheresis should be initiated along with antiviral treatment
Somani et al., 2020^25^	01	Case report		Levetiracetam, lacosamide, Phenytoin, Midazolam	Dyspnea (1/2) Myalgias (1/2 Myoclonic status Epilepticus with coma (MSE) (1/2)	RT-PCR positive	CNS and PNS	Asymptomatic COVID-19. Altered mental status in patients with COVID-19
Toscano et al., 2020^26^	05	Correspondence	COVID-19 positive (4/5)	Intra-venous immunoglobulin (IVIG)	Guillain–Barré syndrome	RT-PCR positive	PNS	First symptoms of Guillain–Barré syndrome is similar to the interval seen with GBS that occurs during or after other infections
Virani et al., 2020^27^	01	Case report	COVID-19 positive	Intra venous immunoglobulin (IVIG) HCQ	Guillain–Barré syndrome	RT-PCR positive	PNS	Author cognizant of the neurological presentation of GBS that is likely associated with SARS-CoV-2 infection
Zhao et al., 2020^28^	01	Case report	COVID-19 positive	Arbidol, Lopinavir, Ritonavir	Guillain–Barré syndrome	RT-PCR positive	PNS	Study showed a possible causal association between Guillain-Barré syndrome and SARS-CoV-2 infection

*NS* nervous system, *HCQ* hydroxy chloroquine, *HCQ* hydroxy-chloroquine, *LPV/RTV* lopinavir/ritonavir, *GBS* Guillain–Barré syndrome.

### Prevalence of nervous system complication/manifestations in COVID-19 positive patients

#### Overall prevalence of central and peripheral nervous system (CNS/PNS) symptoms

**Figure 2:**
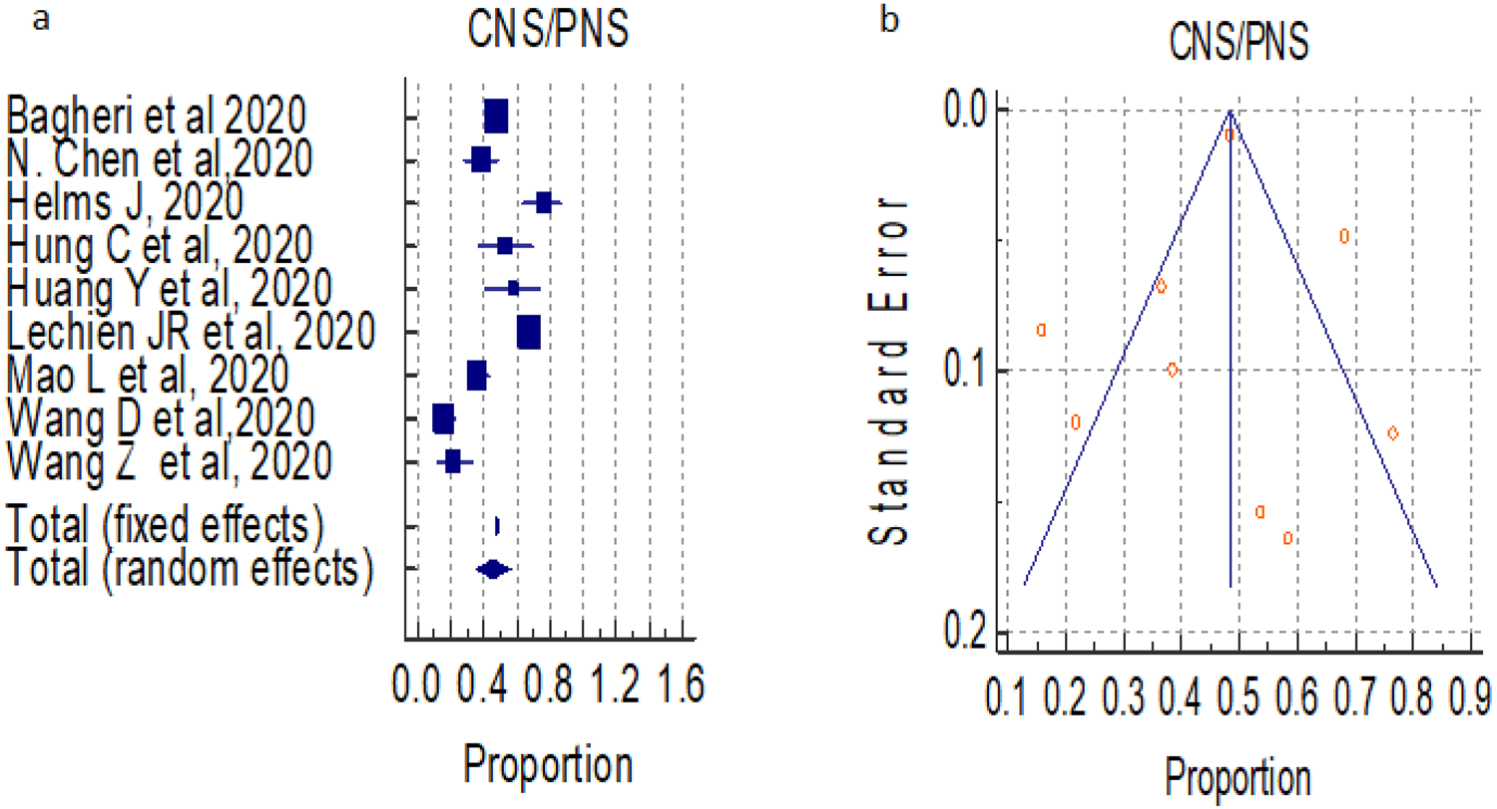
(**a**) Forest plot showed pooled prevalence of CNS-PNS combined among patients with COVID-19 patients. (**b**) Funnel plot showed publication bias among studies evaluating neurological complications of COVID-19.

A total of 09 studies (total 11,147 patients) reported occurrence of CNS-PNS combined symptoms in COVID-19 positive patients, the proportion was 48.278%, 45.718% by fixed and random effect size model, respectively. As there was significant heterogeneity 96.00%, (95% CI for I^2^ 94.08–97.29), we used random effect model. The forest plot is showed in Fig. 2a. No significant publication bias was seen (Fig. 2b).

#### Overall prevalence of nervous system symptoms (CNS)

**Figure 3:**
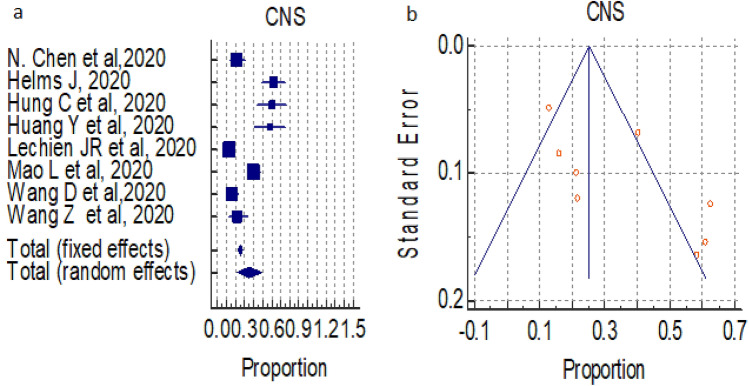
(**a**) Forest plot showed pooled prevalence of CNS complication among patients with COVID-19. (**b**) Funnel plot showed publication bias among studies evaluating neurological complications of COVID-19.

A total of 08 studies (total 1078 patients) reported occurrence of CNS symptoms in COVID-19 positive patients, the proportion was 25.184% and 34.890% by fixed and random effect size model, respectively. As there was significant heterogeneity 95.32%, (95% CI for I^2^ 92.75–96.98) (*P* < 0.0001), we used random effect model. The forest plot is showed in Fig. 3a. Publication bias was seen, may be due to a smaller number of publications (Fig. 3b).

#### Overall prevalence of peripheral nervous system (PNS) symptoms

**Figure 4:**
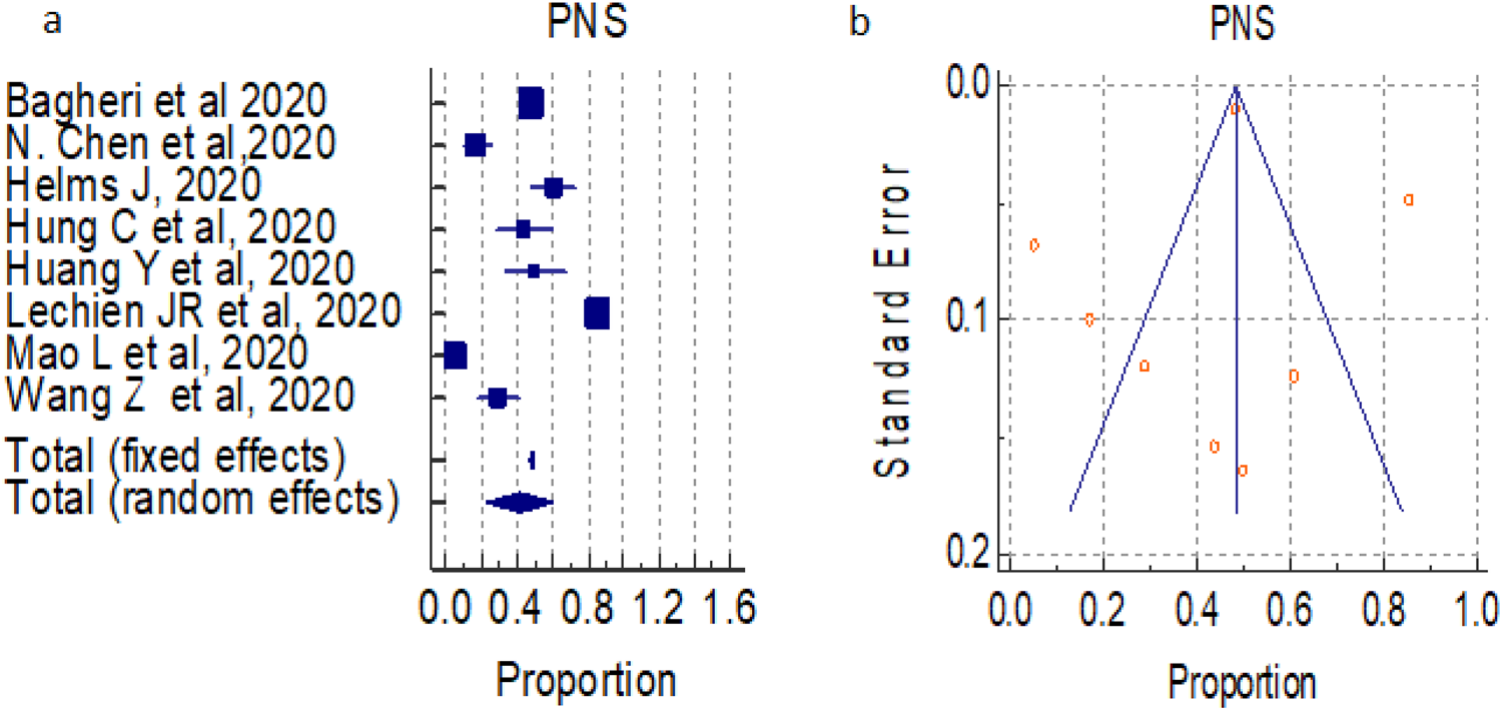
(**a**) Forest plot showed pooled prevalence of PNS complications among patients with COVID-19. (**b**) Funnel plot showed publication bias among studies evaluating neurological complications of COVID-19.

A total of 08 studies (total 11,009 patients) reported occurrence of PNS symptoms in COVID-19 positive patients, the proportion 41.366% and 48.386% by random and fixed effect size model, respectively. As there was significant heterogeneity 98.82% (95% CI for I^2^ 98.43–99.11) (*P* < 0.0001), we used random effect model. The forest plot is showed in Fig. 4a. No significant publication bias was seen (Fig. 4b).

### Prevalence of ANOSMIA/HYPOSMIA as symptoms

**Figure 5:**
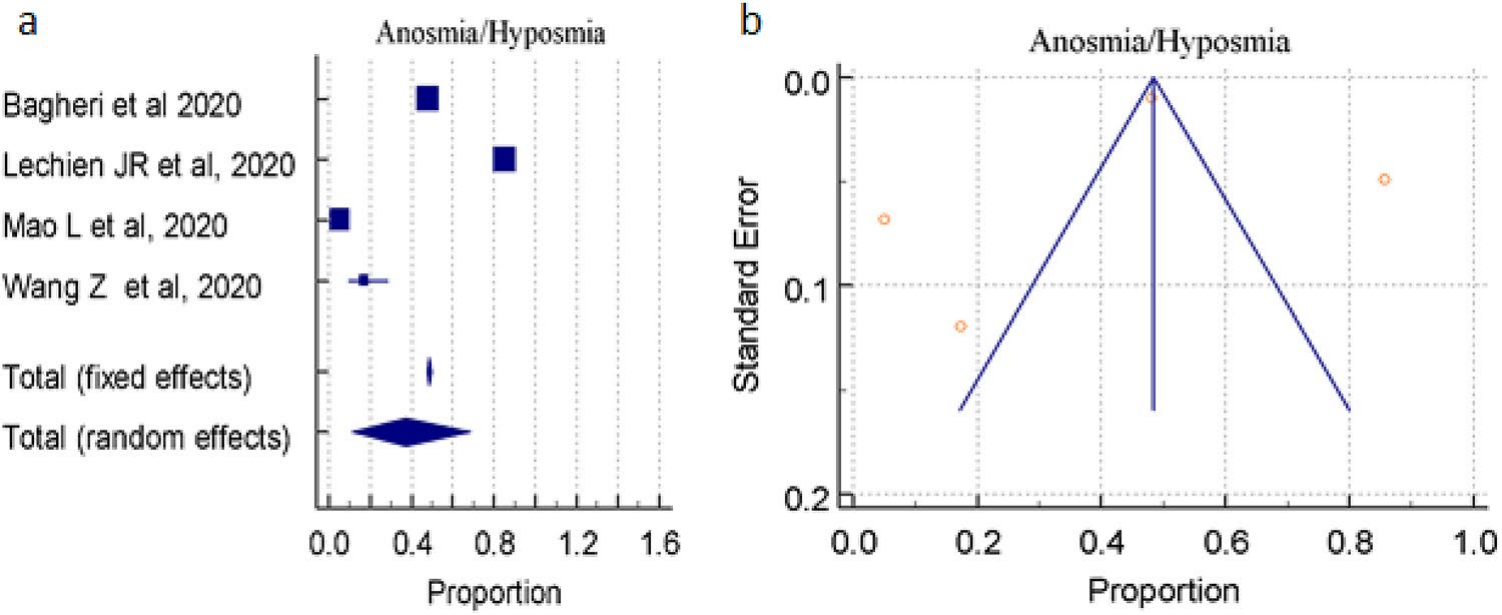
(**a**) Forest plot showed pooled prevalence of anosmia/hyposmia complications among patients with COVID-19. (**b**) Funnel plot showed publication bias among studies evaluating neurological complications of COVID-19.

A total of 3 studies (total 10,769 patients) reported occurrence of anosmia/hyposmia symptoms in COVID-19 positive patients, the proportion 48.547%, 37.270% by random and fixed effect size model, respectively. As there was significant heterogeneity 99.47% (95% CI for I^2^ 99.26–99.61), we used random effect model. The forest plot is showed in Fig. 5a. Publication bias was seen, may be due to very a smaller number of publications. (Fig. 5b).

#### Overall prevalence of Myalgia

**Figure 6:**
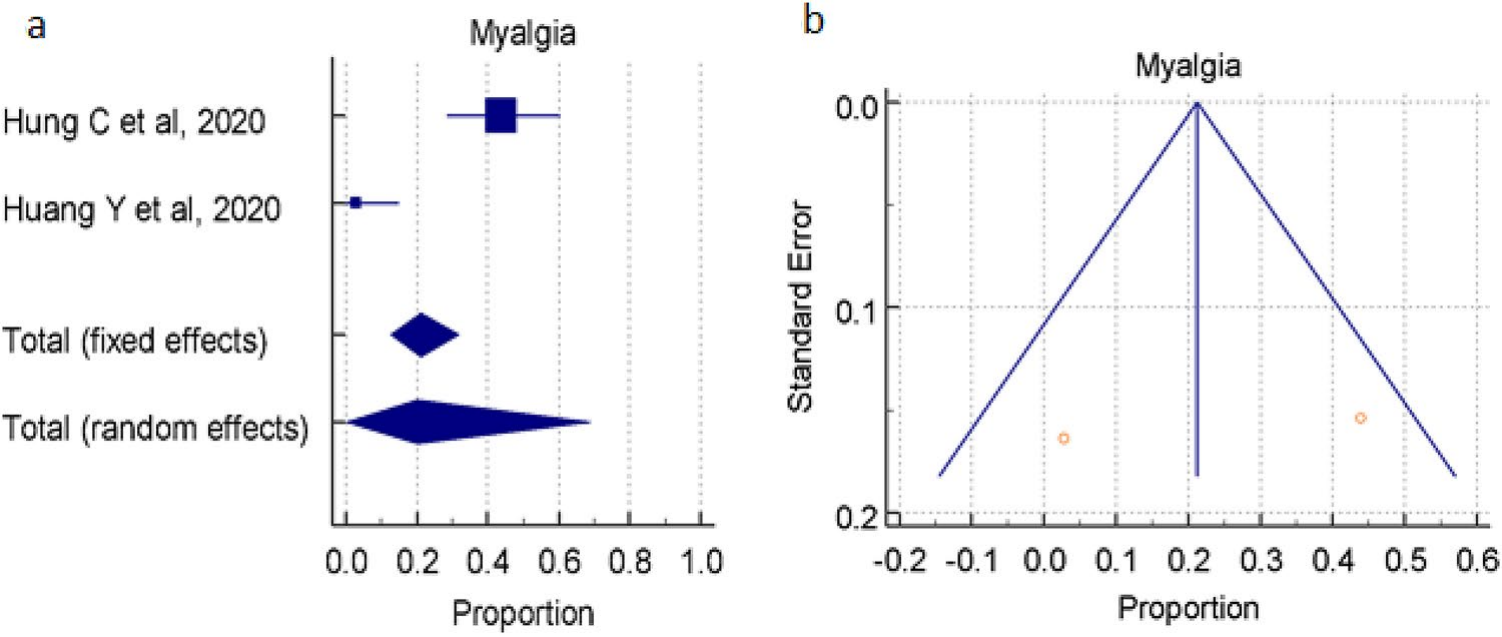
(**a**) Forest plot showed pooled prevalence of Myalgia among patients with COVID-19. Incidence of Myalgia symptoms. (**b**) Funnel plot showed publication bias among studies evaluating neurological complications of COVID-19.

A total of 2 studies (total 77 patients) reported occurrence of Myalgia in COVID-19 positive patients, the proportion 21.279%, 19.994% by random and fixed effect size model, respectively. As there was significant heterogeneity 95.40% (95% CI for I^2^ 86.48–98.44), we used random effect model. The forest plot is showed in Fig. 6a. No significant publication bias was seen (Fig. 6b).

#### Overall prevalence of fatigue

**Figure 7:**
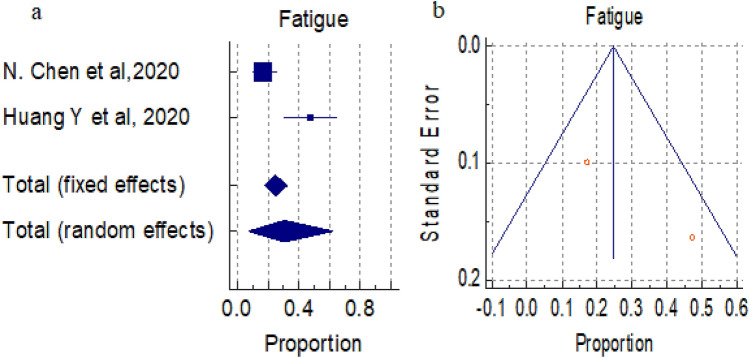
(**a**) Forest plot showed pooled prevalence of Fatigue among patients with COVID-19. (**b**) Funnel plot showed publication bias among studies evaluating neurological complications of COVID-19.

A total of 2 studies (total 135patients) reported occurrence of fatigue in COVID-19 positive patients, the proportion 24.674% and 30.810% by fixed and random effect size model, respectively. As there was significant heterogeneity 91.33% (95% CI for I^2^ 69.43–97.54) (*P* = 0.0007), we used random effect model. The forest plot is showed in Fig. 7a. No significant publication bias was seen (Fig. 7b).

#### Overall prevalence of dyspnea

**Figure 8:**
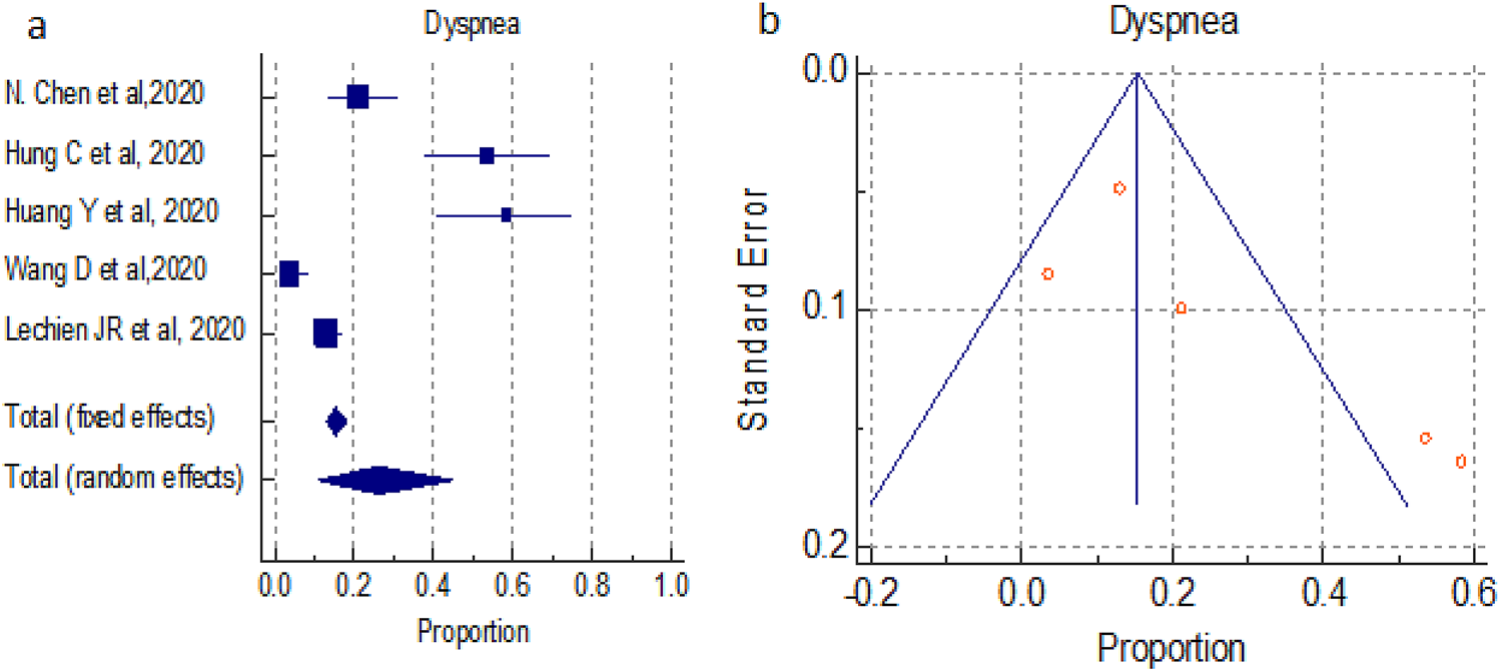
(**a**) Forest plot showed pooled prevalence of Dyspnea among patients with COVID-19. (**b**) Funnel plot showed publication bias among studies evaluating neurological complications of COVID-19.

A total of 5 studies (total 731 patients) reported occurrence of Dyspnea symptoms in COVID-19 positive patients, the proportion 15.419% and 26.131% by fixed and random effect size model, respectively. As there was significant heterogeneity 95.48% (95% CI for I^2^ 92.01–97.44) (*P* < 0.0001), we used random effect model. The forest plot is showed in Fig. 8a. No significant publication bias was seen (Fig. 8b).

#### Overall prevalence of headache

**Figure 9:**
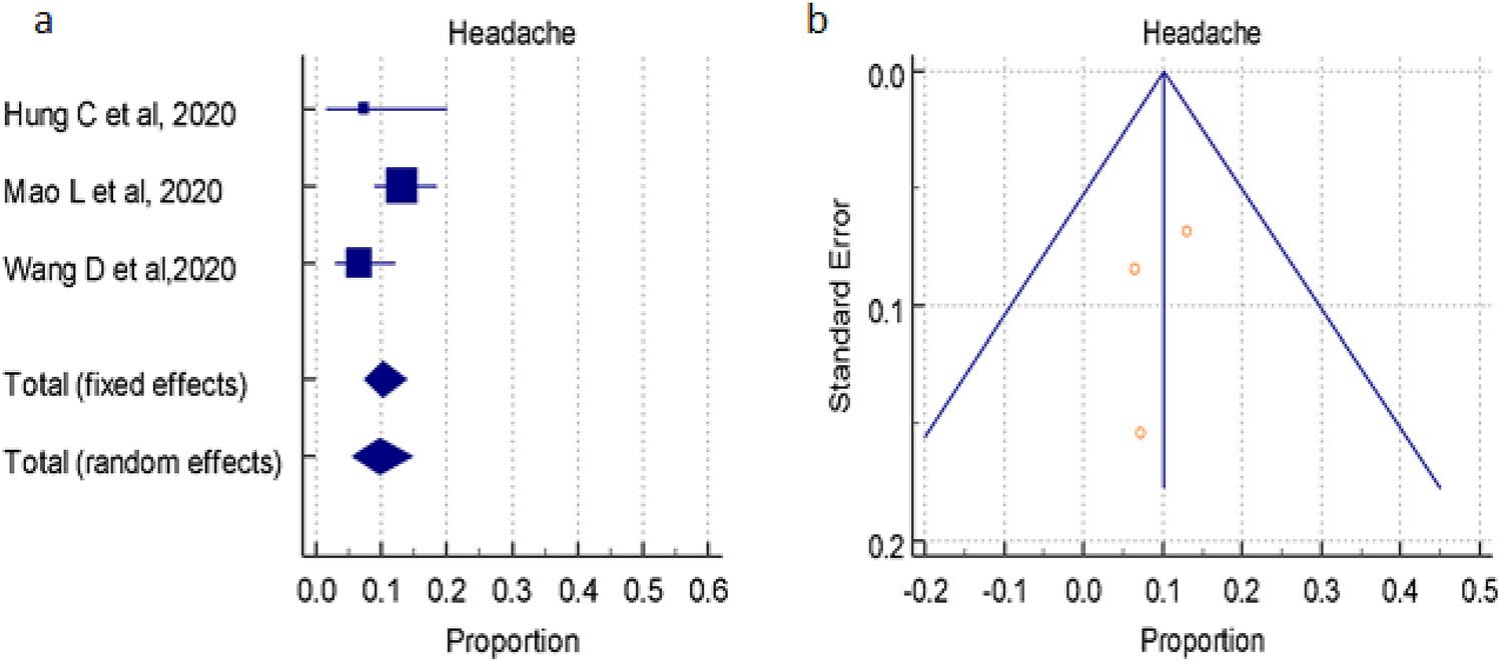
(**a**) Forest plot showed pooled prevalence of Headache among patients with COVID-19. (**b**) Funnel plot showed publication bias among studies evaluating neurological complications of COVID-19.

A total of 3 studies (total 629patients) reported occurrence of Headache in COVID-19 positive patients, the proportion 10.263%, 9.727% by random and fixed effect size model, respectively. As there was not significant heterogeneity 52.06% (95% CI for I^2^ 0.00–86.21), we used random effect model. The forest plot is showed in Fig. 9a. No significant publication bias was seen (Fig. 9b).

#### Overall prevalence of impaired consciousness

**Figure 10:**
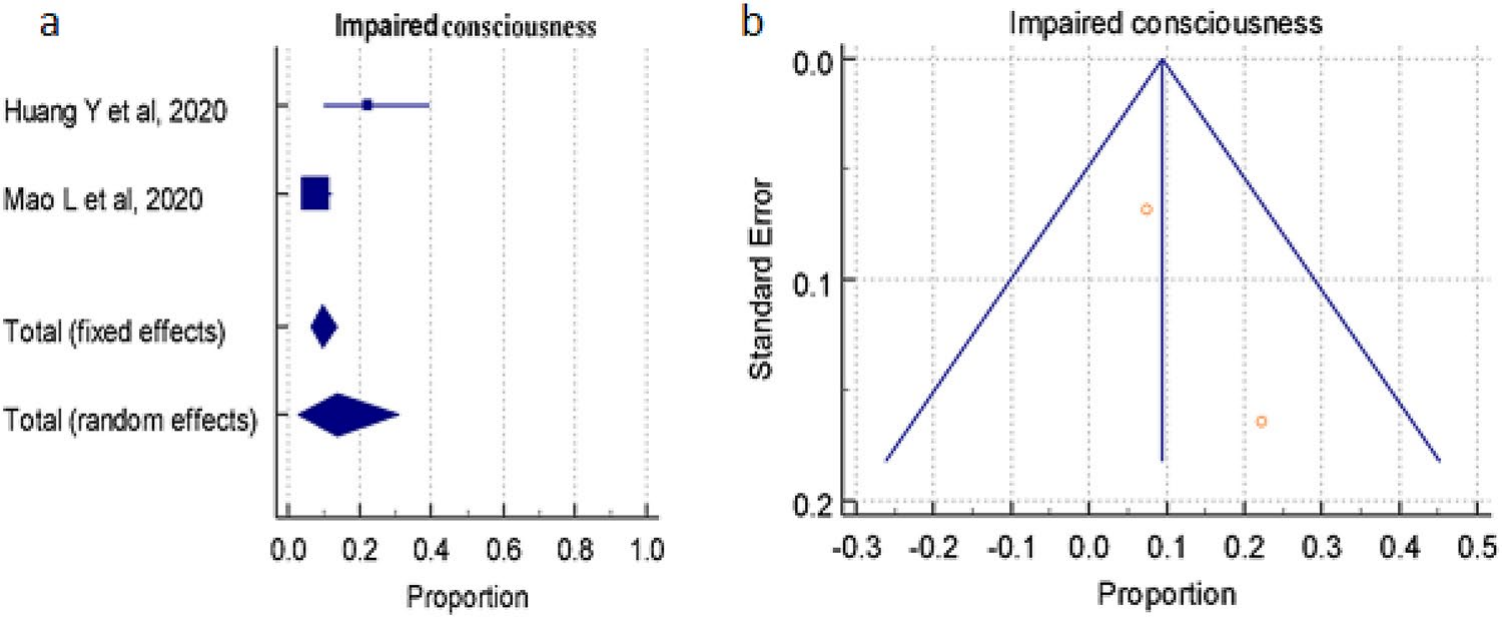
(**a**) Forest plot showed pooled prevalence/incidence of Impaired Consciousness among patients with COVID-19. (**b**) Funnel plot showed publication bias among studies evaluating neurological complications of COVID-19.

A total of 2 studies (total 629patients) reported occurrence of Impaired Consciousness in COVID-19 positive patients, the proportion 9.471%, 13.580% by random and fixed effect size model, respectively. As there was significant heterogeneity 83.49% (95% CI for I^2^ 31.48–96.02), we used random and fixes effect model. The forest plot is showed in Fig. 10a. No significant publication bias was seen (Fig. 10b).

### Network analysis of neurological manifestations

**Figure 11:**
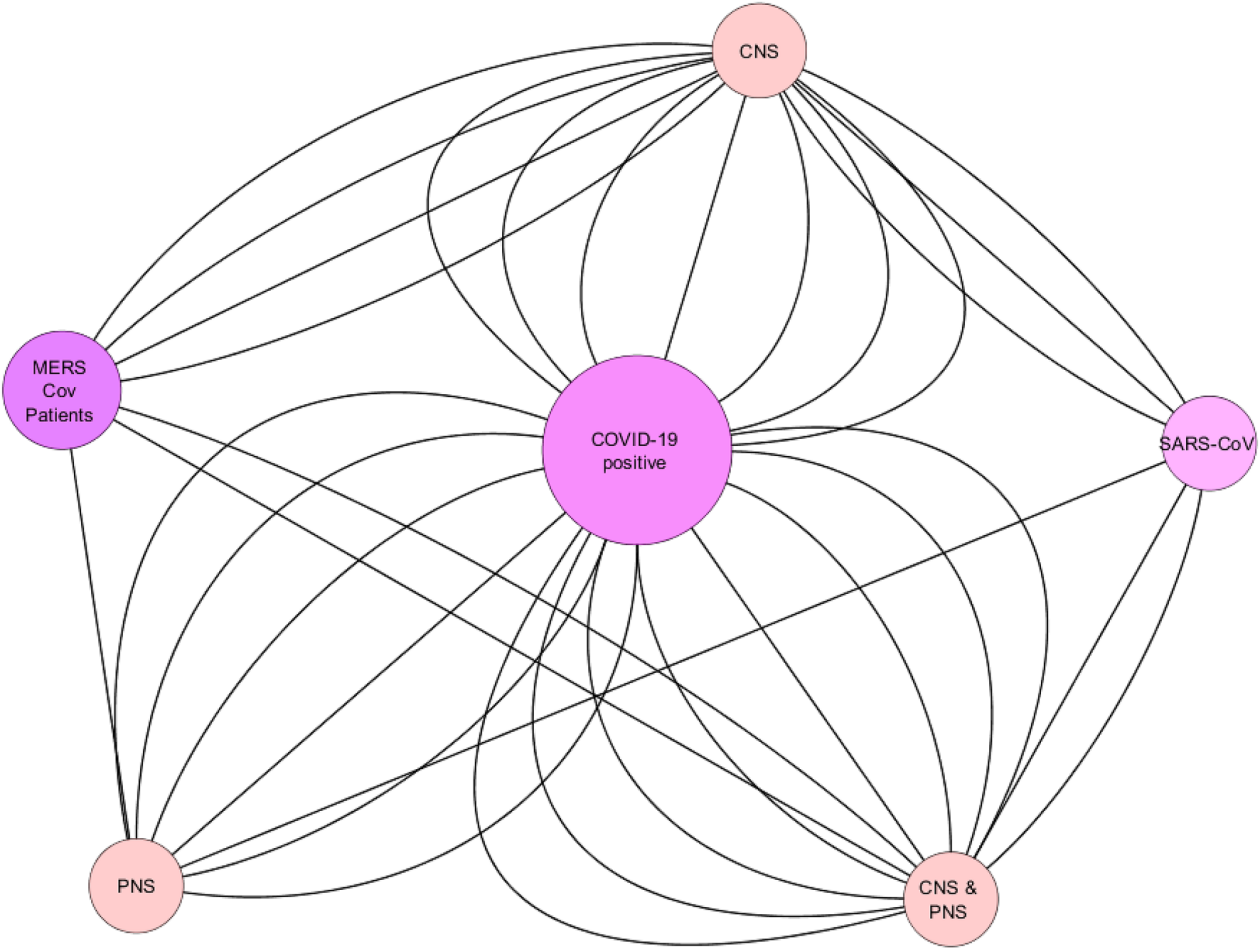
Overall Network analysis which showed the relationship with three main pandemics i.e., SARS-2002, MERS-2012 and COVID-19 with the CNS, PNS or combined manifestations. *Note*: The size of node defines the degree of relation with the individual neurological symptoms and increased number of edges give the increased relation with the respective nodes. (Edges showing directed association, Node size depicts degree of nodes).

**Figure 12:**
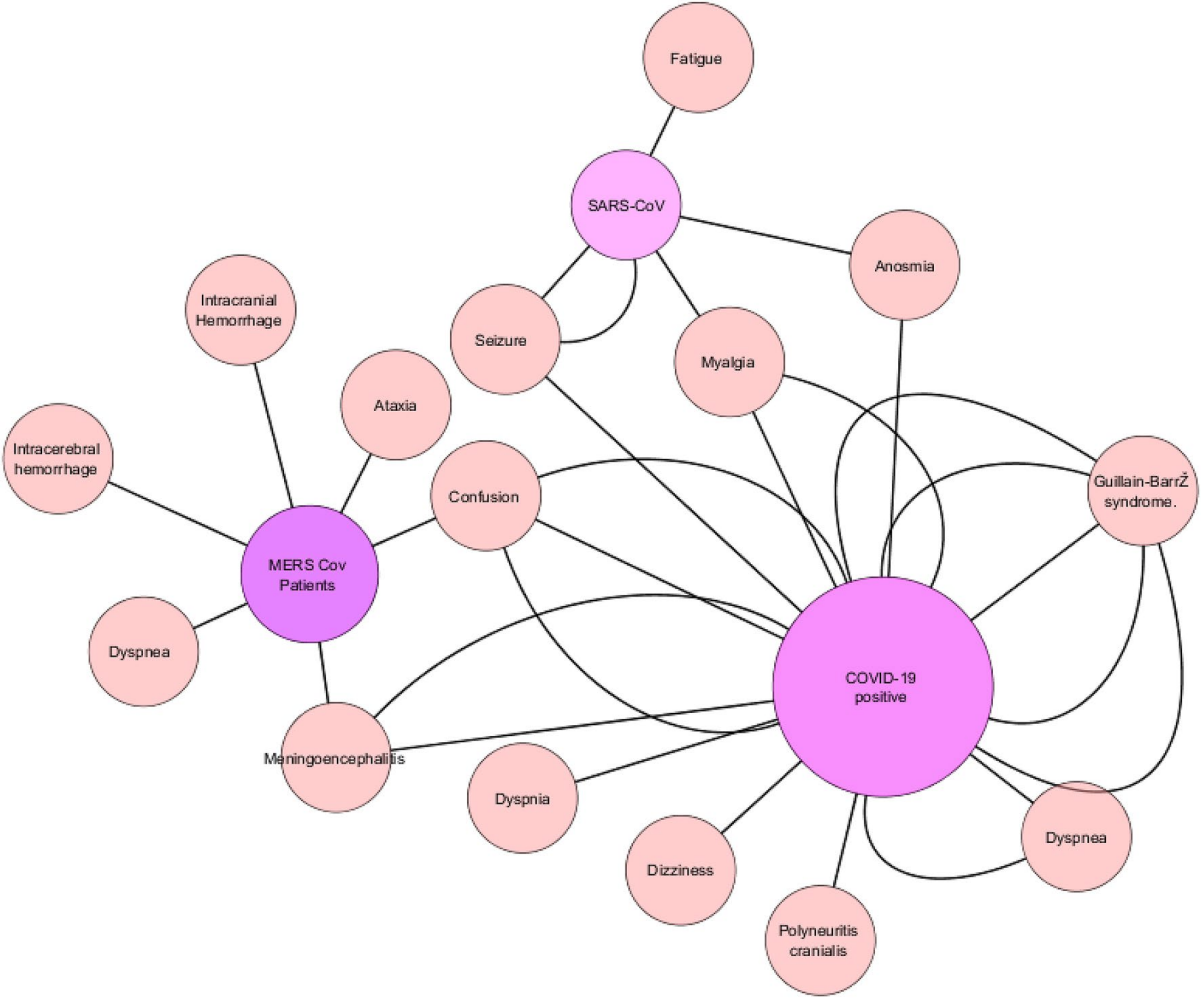
Network analysis showed the impact of neurological manifestation as per described symptoms in case report as well as observational studies. *Note*: The size of node defines the degree of relation with the individual neurological symptoms and increased number of edges give the increased relation with the respective nodes. (Edges showing directed association, Node size depicts degree of nodes).

The network analysis of case reports (n = 103) showed that the neurological manifestation of COVID-19 and other CoV infection is proportionate with each other, whereas, analysis showed that COVID-19 pandemic has more neurological manifestations as compared with other SARS and MERS pandemics in 2002 and 2012 respectively (Fig. 11). The number of studies reported in previous SARS-CoV and MERS-CoV infections were minimal as compared to the COVID-19. The individual neurological manifestation of CNS or PNS ranges from simple headache to the seizure or GBS and even myalgia reported by Lau et al.^32^ and Al-Hameed et al., 2017 reported of GTC (Seizure)^37^ by intracerebral hemorrhage. Kim et al.^36^ reported Guillain–Barré syndrome (GBS), confusion and seizure of several patients. However, recent nCoV-2019 pandemic showed more neurological involvement of meningoencephalitis reported by Duong et al.^18^ and Moriguchi et al.^21^ and Guillain–Barré syndrome was reported by Scheidl et al.^23^, Sedaghat et al.^24^, Toscano et al.^26^, Virani et al.^27^ and Zhao et al.^28^. (Tables 2, 3; Fig. 12).

**Table 3:** Published case reports of previous SARS-CoV and MERS patients of neurological manifestations/ outcomes.

Author, year	Sample size	Type of study	Patient population	Treatment	Neurological condition	2019-nCoV presence and diagnosis	Type of NS involvement (CNS/PNS)		Remarks
**Severe acute respiratory syndrome (SARS-CoV)**									
Chao et al., 2003^29^	01	Case report	SARS-CoV	Broad-spectrum antibiotics and inhalation of Ribavirin	Myalgia (Weakness and numbness in both legs)	chest x-ray (CXR), PCR	PNS		Systemic inflammatory response syndrome, has been proposed to play a role in the nerve damage
Hung et al., 2003^30^	01	Case report	SARS-CoV	Ribavirin Propofol Phenytoin	Seizure	RT PCR, CSF	CNS		First reported the entry of SARS-CoV into the CSF
Hwang et al., 2006^31^	01	Case report	SARS-CoV	Ribavirin, Prednisolone	Anosmia	RT-PCR	PNS		Anosmia persisted during more than 2 years of follow-up
Lau et al., 2004^32^	01	Case report	SARS-CoV	Ribavirin hydrocortisone	Myalgia GTC	RT-PCR CSF-P	CNS and PNS		Generalized convulsion with a positive RT-PCR for SARS-CoV in the CSF suggests possible infection of the central nervous system by SARS-CoV
Umapathi et al., 2004^33^	05	Observational	SARS-CoV	Immunoglobulin (IVIg), Methylprednisolone, Ribavarin, Convalescent serum, LMWH	Loss of consciousness (1/5) Infarction (5/5)		CNS		Out of 206, 05 patients experience neurological manifestations. All five have experience d cerebral infarction
**Middle East respiratory syndrome corona virus (MERS-CoV)**									
Algahtani et al., 2016^34^	02	Observational	MERS-CoV positive	Intravenous hydration, Tazocin, Azithromycin	Headache, Nausea, and vomiting Myalgia, Dizziness Intracerebral hemorrhage	RT-PCR	CNS and PNS	CT showed right frontal lobe intracerebral hemorrhage with massive brain edema and midline shift of 02 patients among 120 patients	
Arabi et al., 2015^35^	03	Observational	MERS-CoV positive	NA	Ataxia (1/3), Vomiting (2/3), Confusion(2/3) Dysmetria (1/3) Dyspnea and hypoxia (1/3) Encephalitis (1/3)	RT-PCR and CSF	CNS and PNS	Brain MRI revealed striking changes characterized by widespread, bilateral hyperintense lesions on T2-weighted imaging within the white matter and subcortical areas of the frontal, temporal, and parietal lobes, the basal ganglia, and corpus callosum	
Al-Hameed et al., 2017^19^	01	Case report	MERS-CoV positive	Peginterferon Alpha-2a, Ribavirin, and Methylprednisolone (i.v.)	Intracerebral hemorrhage Shortness of breath	RT-PCR	CNS	Neurological symptoms associated with MERS-COV	
Kim et al., 2017^36^	04/23	Observational	MERS-CoV positive	Interferon alpha-2a, Ribavirin, and Lopinavir/ Ritonavir	Dyspnea (2/4) Myalgia or arthralgia (2/4) Guillain–Barré syndrome (2/4) Acute sensory neuropathy (3/4) Headache (2/23) Confusion (5/23) Seizure (0/23) Nausea and vomiting (18/23)	RT-PCR	CNS and PNS	GBS, ICU-acquired weakness, or acute sensory neuropathy that resulted from a toxin or infection	
Saad et al., 2014^20^	70	Observational	MERS-CoV positive	NA	Shortness of breath (42/70) Fatigue (29/70) Myalgia or arthralgia (14/70) Vomiting (21/70) Headache (9/70) Confusion (18/70)	RT-PCR		MERS-CoV can cause severe infection in the age ≥ 65 years with more requirement of intensive care and a high mortality	

*NA* not available, *RT-PCR* real-time polymerase chain reaction, *CSF* cerebrospinal fluid, *GTC* generalized tonic–clonic seizure, *GBS* Guillain–Barré syndrome, *i.v.* Intravenous, *CT* computed tomography, *LMWH* Low-molecular-weight heparin.

Gutiérrez-Ortiz et al.^45^ reported the single case of Miller Fisher Syndrome and polyneuritis cranialis, depicting the absence of immune response during COVID-19.

## Discussion

**T**he present systematic review and meta-analysis revealed that the pattern of neurological manifestations of nCoV-2019 is related to the previous two ancestral pandemics i.e., SARS-2002 and MERS-2012. The main clinical manifestations of SARS and MERS were fever, chills, dry cough, and difficulty breathing which is related to nCoV-2019 symptoms as well. In moderate to severe cases, respiratory failure associated with multiple organ involvement may occur^32^. In addition, some of these patients developed neurological manifestations such as encephalitis, polyneuropathy, and aortic ischemic stroke^38^. Autopsy revealed overall deteriorating findings namely infiltration of monocytes and lymphocytes in the vessel wall, ischemic changes of neurons, demyelization of nerve fibers to the cerebral edema and meningeal vasodilation in patients.

Present systematic review and meta-analysis aimed to access the neurological manifestation of nCoV-2019 infection, which can further be classified into central nervous system (CNS) symptoms namely dizziness, headache, impaired consciousness, acute cerebrovascular disease, ataxia, and epilepsy and peripheral nervous system (PNS) symptoms like hypoplasia, hyposmia, neuralgia, and hypogeusia were studied^15,46^.

Ina study by Hung et al.^30^ showed SARS positivity in CSF sample of SARS-CoV and genome sequences of SARS in brain sample. MERS outbreak in 2012–13 also reported delirium, neuropathy and acute cerebrovascular disease^34,36^whereas Saad et al.^20^ reported confusion and seizures in 18 and 6 of the participants, respectively out of 70 MERS patients. Therefore, published literature showed the indication of neurotropism by the CoV family. Recent study by Moriguchi, et al.^21^, showed patients with SARS-CoV-2 suffered from meningitis/encephalitis which were confirmed by RT-PCR detection in cerebrospinal fluid.

The exact reason is unclear, however, few described in the literature are, development of focal meningitis/encephalitis affecting the rhino- or gustatory-cortex or sub-cortical ascending/descending tracts in the CoV positive patients is one of the reasons which may be easily detect viral-RNA in the cerebrospinal fluid (CSF) of infected patients^15,21^. In our network study meningitis/encephalitis, seizure and confusion were well connected to the SARS, MERS and COVID-19 pandemics.

PNS symptoms appeared due to involvement of peripheral nerves, including the cranial nerves which further associate to cranial nerves I, VII, IX, and X by SARS–CoV2^46^. Our study, reported common neurological signs and symptoms were dyspnea (15.419% and 26.131%), fatigue (24.674% and 30.810%), myalgia (19.994–21.279%), loss of consciousness (9.471–13.580%) and agnosia (37.270–48.547%). However, few case reports have reported even more severe neurological manifestations like seizure, GBS or meningitis/encephalopathy (Figs. 11, 12). However, none of the studies observed the viral neurotropism but few authors have reported that the patients with neurological manifestations have the positive-CSF of COVID-19^39^. Therefore, it is very early to explain whether neurological dysfunction is due to direct viral injury or systemic disease.

The other school of thought is that the neurotropism of CoV family occur mainly through two pathways, i.e., the hematogenous and retrograde route through respiratory infection. An experimental study showed that MERS-CoV tissue pantropism occurs after the viral entry into blood stream through endothelial infection in the choroid plexus^7,22^.

High mortality and fast spread of the CoV-family viruses occurs due to the high fusion of these virus and rapid replication involving angiotensin converting enzyme-2 (ACE-2) receptor which is present throughout the vital organs^16^. Therefore, presence of ACE 2 receptor in brain is an important factor and link to study the neurological manifestations in 2019-nCoV infection. Patel AB, 2020^40^ described that there is evidence for a major involvement of excessive brain ACE/Ang II/ AT-1R axis leading to increased activation of oxidative stress, apoptosis and neuroinflammation causing neurodegeneration and other brain disorders^44^. At last, the probable chance of neurological manifestation may be due to the cytokines overload and reduced immunity. Immuno-inflammation plays an important role in viral infection and which leads to the systemic inflammatory response syndrome (SIRS)^41^. In vitro study suggested that primary glial cells release large number of inflammatory cytokines namely IL-6, IL-12, IL-15, and TNF-α after being infected with SARS-CoV^42^. Further, study suggests that virus-induced SIRS or SIRS-like immune disorders may cause mortality due to involvement of macrophages, microglia, and astrocytes in the CNS^43^.

## Concluding remarks

The nCoV-2019 infection starts with mild flu-like symptoms to the late neurological manifestations in moderate to severely COIVD-19 positive patients. However, patients with comorbid conditions namely lung diseases, diabetes, obesity, hypertension, cardiac or kidney disease are more prone to exacerbation of disease or neurological manifestations or death. Further, studies suggested that the involvement of both peripheral and central nervous systems, also suggested of neurotropism of nCoV-2019. Therefore, we need more studies to conclude the neuro-invasion in brain and affected areas for the management of patients, worldwide.

## Limitations of the study

In the present study, meta-analysis is restricted to the evaluations of observational and case report studies which was available at the earliest during the synthesis of the current study in the scientific community. Limited studies and rapid spreading nCoV-2019 pandemic restrict the evidence-based studies like randomized control trial (RCT)/double blind studies during the pandemics. Currently, there is other variant new strain is also identified and being studied. Therefore, there may chances of some new neurological symptoms to be reported. The good designed studied during pandemic is the major limitations of systematic analysis or meta-analysis study.

## Abbreviations

SARS: Severe acute respiratory syndrome
MERS: Middle East respiratory syndrome
ARDS: Serious respiratory distress syndrome
COVID-19: Coronavirus disease 19
nCoV-2019: Corona virus 2019
SIRS: Systemic inflammatory response syndrome
RT-PCR: Real time polymerase chain reaction

## References

[1] Prajapat M (2020). Drug targets for corona virus: A systematic review. Indian J Pharmacol.

[2] Huang C (2020). Clinical features of patients infected with 2019 novel coronavirus in Wuhan, China. Lancet.

[3] Lauer SA (2020). The incubation period of coronavirus disease 2019 (COVID-19) from publicly reported confirmed cases: Estimation and application. Ann Intern Med.

[4] Kwong KCNK, Mehta PR, Shukla G, Mehta AR (2020). COVID-19, SARS and MERS: A neurological perspective. J Clin Neurosci.

[5] COVID-19 coronavirus pandemic. https://www.worldometers.info/coronavirus/. Assessed 26 May 2020.

[6] WHO. Coronavirus disease (COVID-19) pandemic; 2020. https://www.who.int/emergencies/diseases/novel-coronavirus-2019/events-as-they-happen. Assessed 26 May 2020.

[7] Li YC, Bai WZ, Hashikawa T (2020). The neuroinvasive potential of SARS-CoV2 may play a role in the respiratory failure of COVID-19 patients. J Med Virol.

[8] Karimi N, Sharifi RA, Rouhani N (2020). Frequent convulsive seizures in an adult patient with COVID-19: A case report. Iran Red Crescent Med J.

[9] Koyuncu OO, Hogue IB, Enquist LW (2013). Virus infections in the nervous system. Cell Host Microbe.

[10] Bagheri SH (2020). Coincidence of COVID-19 epidemic and olfactory dysfunction outbreak. Medrxiv.

[11] Chen N, Zhou M, Dong X (2020). Epidemiological and clinical characteristics of 99 cases of 2019 novel coronavirus pneumonia in Wuhan, China: a descriptive study. Lancet.

[12] Helms J (2020). Neurologic features in severe SARS-CoV-2 infection. N Engl J Med.

[13] Huang Y (2020). Clinical characteristics of 36 non-survivors with COVID-19 in Wuhan, China. MedRxiv.

[14] Lechien JR (2020). Olfactory and gustatory dysfunctions as a clinical presentation of mild-to-moderate forms of the coronavirus disease (COVID-19): a multicenter European study. Eur Arch Otorhinolaryngol.

[15] Mao L (2020). Neurological manifestations of hospitalized patients with COVID-19 in Wuhan, China: a retrospective case series study. JAMA Neurole.

[16] Wang D (2020). Clinical characteristics of 138 hospitalized patients with 2019 novel coronavirus-infected pneumonia in Wuhan, China. JAMA.

[17] Wang Z (2020). Clinical features of 69 cases with coronavirus disease 2019 in Wuhan, China. Clin Infect Dis.

[18] Duonga L, Xub P, Liua A (2020). Meningoencephalitis without respiratory failure in a young female patient with COVID-19 infection in Downtown Los Angeles, early April 2020. Brain Behav Immun.

[19] Al-Hameed FM (2017). Spontaneous intracranial hemorrhage in a patient with Middle East respiratory syndrome corona virus Fahad M. Saudi Med J.

[20] Saad M (2014). Clinical aspects and outcomes of 70 patients with Middle East respiratory syndrome coronavirus infection: a single-center experience in Saudi Arabia. Int J Infect Dis.

[21] Moriguchi T (2020). A first case of meningitis/encephalitis associated with SARS-Coronavirus-2. Int J Infect Dis.

[22] Paniz-Mondolfi A (2020). Central nervous system involvement by severe acute respiratory syndrome coronavirus-2 (SARS-CoV-2). J Med Virol.

[23] Scheidl E, Canseco DD, Hadji-Naumov A, Bereznai B (2020). Guillain-Barré syndrome during SARS-CoV-2 pandemic: A case report and review of recent literature. J Peripher Nerv Syst.

[24] Sedaghat Z, Karimi N (2020). Guillain Barre syndrome associated with COVID-19 infection: A case report. J Clin Neurosci.

[25] Somani S, Pati S, Gaston T, Chitlangia A, Agnihotri S (2020). De novo status epilepticus in patients with COVID-19. Ann Clin Transl Neurol.

[26] Toscano G (2020). Guillain-Barré syndrome associated with SARS-CoV-2. N Engl J Med.

[27] Virani A (2020). Guillain-Barré syndrome associated with SARS-CoV-2 infection. ID Cases.

[28] Zhao H, Shen D, Zhou H, Liu J, Chen S (2020). Guillain-Barré syndrome associated with SARS-CoV-2 infection: Causality or coincidence?. Lancet Neurol.

[29] Chao CC (2003). Peripheral nerve disease in SARS: Report of a case. Neurology.

[30] Hung EC (2003). Detection of SARS coronavirus RNA in the cerebrospinal fluid of a patient with severe acute respiratory syndrome. Clin Chem.

[31] Hwang CS (2006). Olfactory neuropathy in severe acute respiratory syndrome: Report of a case. Acta Neurol Taiwan.

[32] Lau KK, Yu WC, Chu CM, Lau ST, Sheng B, Yuen KY (2004). Possible central nervous system infection by SARS coronavirus. Emerg Infect Dis.

[33] Umapathi T (2004). Large artery ischaemic stroke in severe acute respiratory syndrome (SARS). J Neurol.

[34] Algahtani H, Subahi A, Shirah B (2016). Neurological complications of middle east respiratory syndrome coronavirus: A report of two cases and review of the literature. Case Rep Neurol Med.

[35] Arabi YM (2015). Severe neurologic syndrome associated with Middle East respiratory syndrome corona virus (MERS-CoV). Infection.

[36] Kim JE (2017). Neurological complications during treatment of middle east respiratory syndrome. J Clin Neurol.

[37] Liberati A (2009). The PRISMA statement for reporting systematic reviews and meta-analyses of studies that evaluate health care interventions: Explanation and elaboration. BMJ.

[38] Tsai LK (2004). Neuromuscular disorders in severe acute respiratory syndrome. Arch Neurol.

[39] Conde CG, Quintana PLD, Quintero MID, Ramos VY, Moscote SLR (2020). Neurotropism of SARS-CoV 2: Mechanisms and manifestations. J Neurol Sci.

[40] Patel VB, Zhong JC, Grant MB, Oudit GY (2016). Role of the ACE2/Angiotensin 1–7 axis of the renin-angiotensin system in heart failure. Circ Res.

[41] Mehta P (2020). COVID-19: Consider cytokine storm syndromes and immunosuppression. Lancet.

[42] Bohmwald K, Gálvez NMS, Ríos M, Kalergis AM (2018). Neurologic alterations due to respiratory virus infections. Front Cell Neurosci.

[43] Yin CH, Wang C, Tang Z, Wen Y, Zhang SW, Wang BE (2004). Clearance effect of different blood purification techniques on parathyroid hormone in renal function failure patients on maintenance hemodialysis. Zhongguo Wei Zhong Bing Ji Jiu Yi Xue.

[44] Sarma P, Prajapat M, Avti P, Kaur H, Kumar S, Medhi B (2020). Therapeutic options for the treatment of 2019-novel coronavirus: An evidence-based approach. Indian J Pharmacol.

[45] Gutiérrez-Ortiz C, Méndez A, Rodrigo-Rey S, San Pedro-Murillo E, Bermejo-Guerrero L, Gordo-Mañas R, de Aragón-Gómez F, Benito-León J (2020). Miller Fisher Syndrome and polyneuritis cranialis in COVID-19. Neurology.

[46] Finsterer J, Stollberger C (2020). Causes of hypogeusia/hyposmia in SARS-CoV2 infected patients. J Med Virol.

